# Electrolytic Sulfuric Acid Production with Carbon Mineralization for Permanent Carbon Dioxide Removal

**DOI:** 10.1021/acssuschemeng.2c07441

**Published:** 2023-03-13

**Authors:** Laura N. Lammers, Yanghua Duan, Luis Anaya, Ayumi Koishi, Romario Lopez, Roxanna Delima, David Jassby, David L. Sedlak

**Affiliations:** † Department of Environmental Science, Policy, and Management, University of California, Berkeley, California 94720, United States; ‡ Travertine Technologies, Inc., Boulder, Colorado 80301, United States; § Department of Civil and Environmental Engineering, University of California, Berkeley, California 94720, United States; ∥ Energy Geoscience Division, Lawrence Berkeley National Laboratory, Berkeley, California 94720, United States; ⊥ Department of Civil and Environmental Engineering, University of California, Los Angeles, California 90095, United States

**Keywords:** carbon dioxide removal (CDR), carbon mineralization, chemical waste upcycling, sulfuric acid, critical element extraction

## Abstract

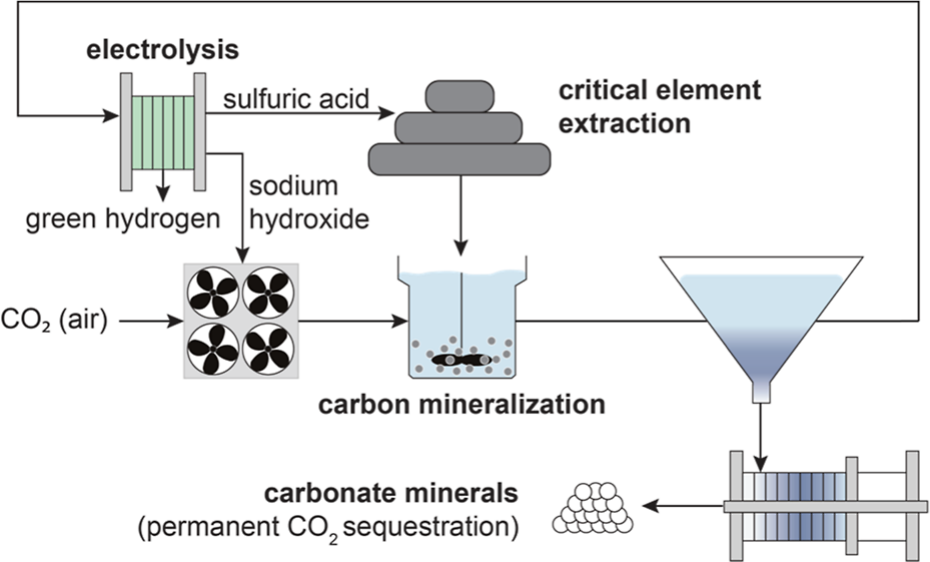

Several billion metric tons per year of durable carbon dioxide removal (CDR) will be needed by mid-century to prevent catastrophic climate warming, and many new approaches must be rapidly scaled to ensure this target is met. Geologically permanent sequestration of carbon dioxide (CO_2_) in carbonate minerals—carbon mineralization—requires two moles of alkalinity and one mole of a CO_2_-reactive metal such as calcium or magnesium per mole of CO_2_ captured. Chemical weathering of geological materials can supply both ingredients, but weathering reactions must be accelerated to achieve targets for durable CDR. Here, a scalable CDR and mineralization process is reported in which water electrolysis is used to produce sulfuric acid for accelerated weathering, while a base is used to permanently sequester CO_2_ from air into carbonate minerals. The process can be integrated into existing extractive processes by reacting produced sulfuric acid with critical element feedstocks that neutralize acidity (e.g., rock phosphorus or ultramafic rock mine tailings), with calcium- and magnesium-bearing sulfate wastes electrolytically upcycled. The highest reported efficiency of electrolytic sulfuric acid production is achieved by maintaining catholyte feed conditions that minimize Faradaic losses by hydroxide permeation of the membrane-separated electrochemical cell. The industrial implementation of this process provides a pathway to gigaton-scale CO_2_ removal and sequestration during the production of critical elements needed for decarbonizing global energy infrastructure and feeding the world.

## Abstract 2

Extraction of critical elements from feedstocks using electrolytically recycled acid enables the scalable removal of atmospheric carbon dioxide with permanent mineralization.

## Introduction

Global anthropogenic carbon dioxide (CO_2_) emissions are approximately 50 gigatons per year, and affordable solutions to durably sequester CO_2_ are needed to prevent catastrophic climate change.^1,2^ Recent IPCC projections indicate that around 6 billion metric tons (Gt) per year of direct air capture of CO_2_ with durable storage (DACS) are required to reduce atmospheric CO_2_ concentrations to levels that safely limit global warming.^3^ To match the large scale of global CO_2_ emissions, scientists have turned to natural processes for inspiration. Formation of carbonate minerals represents a safe, stable, and geologically permanent way to remove and sequester CO_2_,^4,5^ but mineral carbonation requires both a source of CO_2_-reactive elements (e.g., calcium and magnesium) and a permanent sink for acidity (i.e., an alkaline material). Over geologic timescales, the weathering of silicate rocks at Earth’s surface supplies the ingredients for mineral carbonation to regulate the global atmospheric CO_2_ concentration,^6,7^ in a process known as the Urey cycle (Supporting Information). Importantly, the Urey cycle is driven by the ability of rock-forming minerals to neutralize acid, as measured by their acid neutralizing potential (ANP), resulting in CO_2_ dissolution into water and the subsequent precipitation of solid carbonate minerals.

Achieving cost-effective CDR that can be scaled to gigatons per year of CO_2_ sequestration poses a major technological challenge. Direct air capture of CO_2_ is energy-intensive, and many leading direct air capture technologies require several gigajoules of energy—often as heat—to sequester one ton of CO_2_.^8,9^ Production of valuable co-products, such as cement products,^10−12^ or coupling carbon removal with existing extractive processes^13^ can help improve the economic viability of CDR. The mining industry processes billions of tons of rock every year to extract critical elements for electrified energy generation and storage (e.g., nickel, copper, and lithium) and for the fertilizer industry (e.g., phosphorus). Of the billions of tons of tailings and waste rock produced, around 420 Mt consist of basic or ultramafic rocks that have the potential to sequester ∼175 Mt of CO_2_ annually.^14,15^ Carbon dioxide sequestration by mine tailings carbonation has significant room for growth considering that nickel mining will need to increase by more than 5 times by 2040 in order to meet global renewable energy goals,^16^ and tailings production will increase at least in proportion to critical element production as ore grades decrease. Tailings reprocessing can also recover critical elements left behind in mine wastes,^17^ so coupling tailings carbonation with critical element recovery can mitigate the cost of CDR.

Natural rock weathering reactions are too slow to abate human emissions, so many physical and chemical approaches have been developed to accelerate the rate of tailings weathering for CDR.^13,18−23^ Chemical “pH-swing” methods combining acid-accelerated primary silicate mineral dissolution with subsequent base addition can drive rapid carbon mineralization over timescales of hours to days^13,24−27^ because silicate weathering reaction rates increase exponentially with decreasing pH.^28^ The addition of a strong acid such as sulfuric or hydrochloric acid to mafic and ultramafic silicate tailings accelerates weathering and can produce neutralized leachates that minimize heavy metal leaching.^29^ The overall reaction for strong acid-enhanced ultramafic weathering of a representative mineral, forsterite, is given
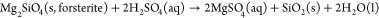
1

Subsequent carbonation of acid-leached magnesium requires the stoichiometric addition of alkalinity to generate solid carbonates,^25,26^ for example,
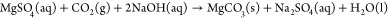
2

Such pH-swing approaches have been shown to effectively accelerate the weathering and carbonation of a variety of tailings and waste rock feedstocks, but the overall process is usually net carbon emitting, given the need for stoichiometric quantities of acid and base.^25^

Rock phosphorus represents another major geological alkalinity source that is largely overlooked in the mineral carbon sequestration literature. Production of phosphoric acid (H_3_PO_4_) for fertilizer consumes around 60% of the global sulfuric acid supply and generates 200–300 Mt of waste gypsum (i.e., phosphogypsum or PG) annually.^30^ Phosphogypsum has been suggested as a feedstock for permanent mineral carbon sequestration, but this process requires 2 equiv of alkalinity per mole of gypsum converted to calcium carbonate.^31−36^ Like enhanced rock weathering, rock phosphorus processing neutralizes sulfuric acid to produce a weak acid, phosphoric acid, by the reaction
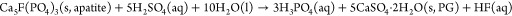
3

In alkaline solutions containing a strong base such as NaOH, the produced solid PG can be readily converted into carbonate minerals
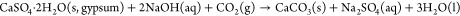
4

Large-scale replacement of gypsum with carbonate minerals has been observed in rock formations.^37^ Importantly, the replacement reaction (reaction 4) is not passivating: with sufficient carbonate alkalinity, conversion of solid gypsum to solid calcium carbonate can proceed rapidly to completion.^33,36,38^ If CO_2_ can be mineralized on an equimolar basis with gypsum consumption, the ANP of rock P consumed during phosphate fertilizer production can theoretically sequester 50–75 Mt/y CO_2_ today.

**Figure 1:**
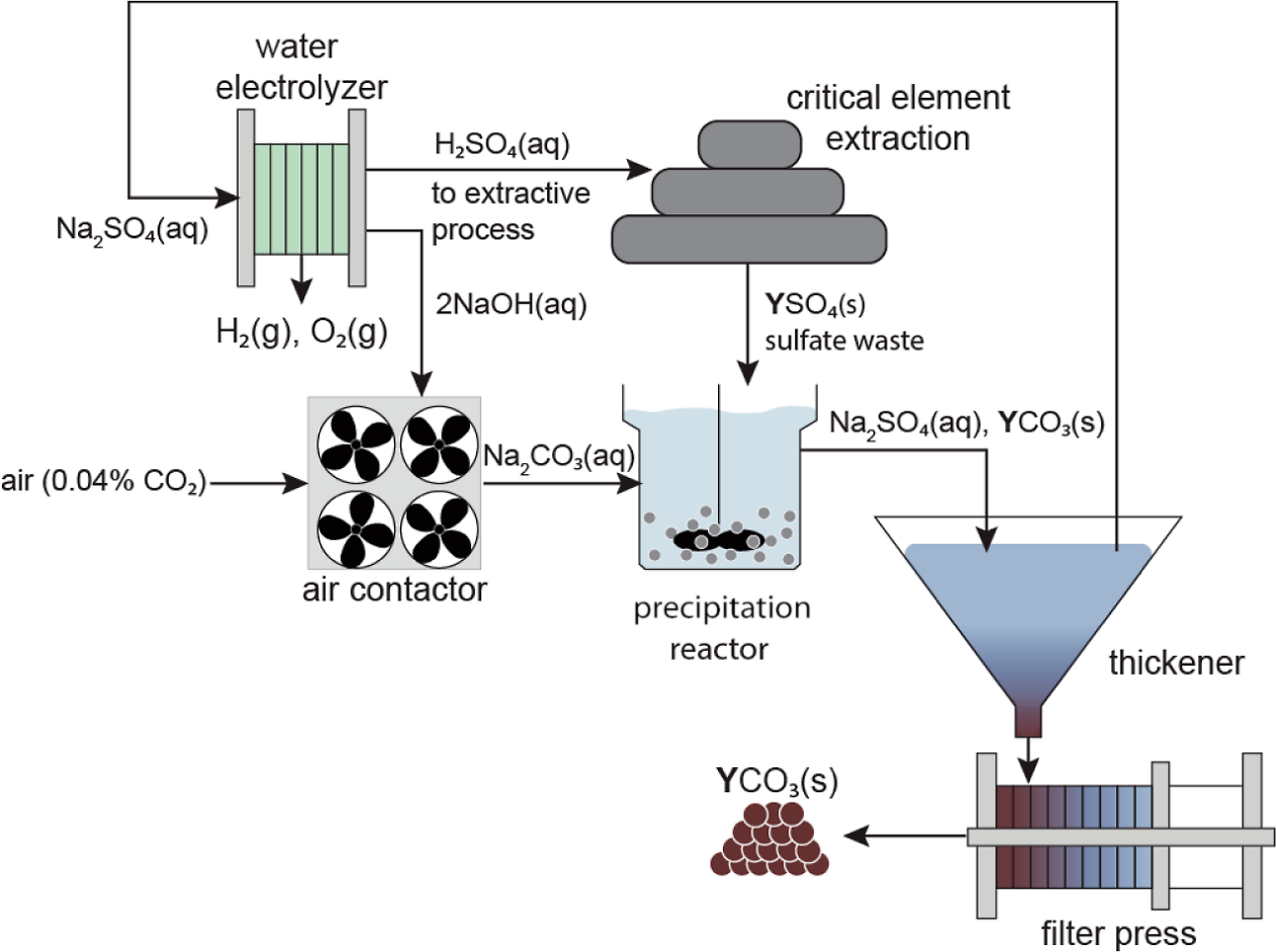
Proposed process for durable CO_2_ sequestration and sulfate waste upcycling. Sulfate solutions fed to an electrochemical cell stack generate sulfuric acid, base solution, hydrogen, and oxygen. The produced acid is used in a critical element extraction process, such as phosphoric acid production from rock phosphorus or critical element extraction from ultramafic silicate materials. These extractive processes generate neutralized sulfate wastes (**Y**SO_4_, where, for example, **Y** = Mg and Ca). In parallel, the base solution produced in the water electrolyzer reacts with CO_2_ from the air to produce carbonate solutions, which combine with the calcium- or magnesium-bearing sulfate waste to precipitate solid carbonate minerals, which are recovered by solid–liquid separation. Aqueous sulfate solutions are recycled to the water electrolyzer to continue the process.

Here, we present an efficient process for CO_2_ mineralization with electrolytic sulfuric acid production (Figure 1) that addresses the large acid and base requirements associated with pH-swing CDR. Inputs to the process include sulfate wastes from sulfuric acid leaching of geological materials with substantial ANP, CO_2_ from the air, and electricity, and products of the process include aqueous sulfuric acid, solid carbonate minerals, and hydrogen gas. The proof-of-concept for the process is established using an integrated bench-scale system that combines a two-compartment, anion-exchange membrane (AEM)-separated electrolysis cell with a precipitation reactor. The tested process is described by the overall reaction
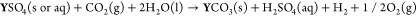
5where **Y** = Ca or Mg. Although the idea of coupling electrochemical acid/base production with CDR is not new,^39−44^ the integrated process developed here achieves the highest reported efficiency by maintaining a low concentration of OH^–^ in the catholyte, reducing Faradaic losses, while at the same time protecting the AEM from degradation in concentrated base.^45^ These improvements allow us to achieve chemical production efficiencies on par with the industrialized chlor-alkali process, enabling substantial net removal of CO_2_ from the air. We evaluate the potential for deploying this process at scale and identify the research needed to enable its use for large-scale CO_2_ sequestration.

## Materials and Methods

### Integrated Reactor Design and Operation

**Figure 2:**
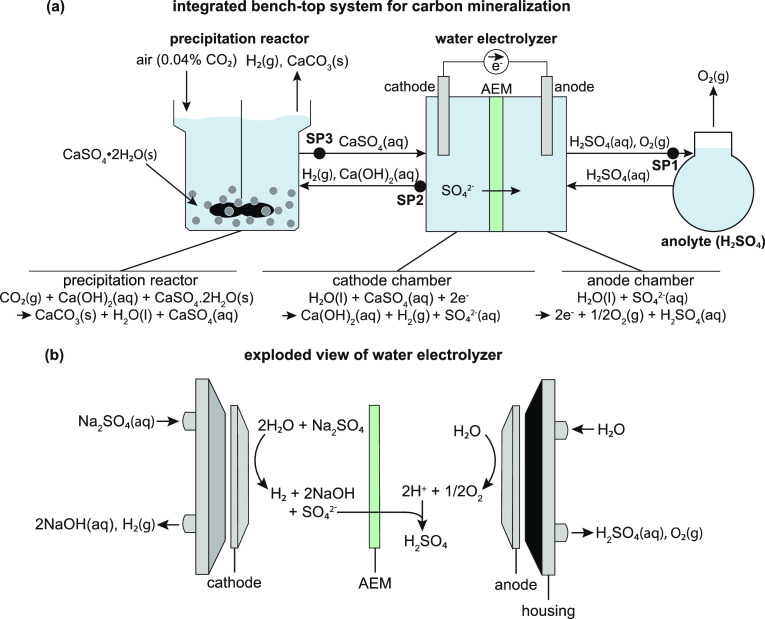
(a) Proof-of-concept integrated carbon mineralization and sulfuric acid production system tested in this study. Air (containing 0.04% CO_2_) is sparged directly into a precipitation reactor filled with a slurry of ground solid gypsum (CaSO_4_·2H_2_O). In the precipitation reactor, CO_2_ reacts with calcium hydroxide (Ca(OH)_2_) produced in the water electrolyzer to form calcium carbonate (CaCO_3_) precipitates. Samples were taken from three sample points (SP1 = anolyte, SP2 = catholyte, and SP3 = precipitation reactor effluent) during experiments. (b) Schematic of electrolyzer reactions, assuming the sulfate source is Na_2_SO_4_(aq).

A bench-top integrated system was developed to establish the proof-of-concept for electrolytic sulfuric acid production with CDR and carbonate mineralization, as illustrated in Figure 2a. In this system, an AEM-separated, finite-gap electrolyzer cell was connected to a mixed-flow precipitation reactor using flexible tubing and peristaltic pumps. The precipitation reactor was supplied with a slurry of milled gypsum in gypsum-equilibrated water. Reactor filtrate was supplied to the cathode chamber of the electrolyzer, where the base was produced by water reduction on the cathode, forming an aqueous calcium hydroxide solution as well as hydrogen gas
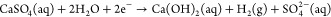
6

Although aqueous calcium sulfate was supplied as the source of sulfate to the electrolyzer in these integrated experiments, the source of sulfate to the electrolyzer can include other salts: **X**_2/***m***_SO_4_(aq), where **X**^***m*+**^ = Na^+^, Ca^2+^, Mg^2+^, etc. Liberated sulfate ions cross the AEM to the anode chamber, where protons and oxygen are generated by water oxidation at the anode to produce sulfuric acid
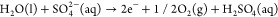
7

The anode solution was recirculated in batches to accumulate sulfuric acid over time. To capture CO_2_, the calcium hydroxide solution produced in the cathode chamber was returned to the precipitation reactor, where it reacted with the calcium sulfate feedstock (gypsum) and CO_2_ introduced by bubbling with air
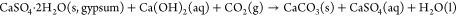
8

Under these conditions, the replacement of gypsum by calcium carbonate is thermodynamically favorable and lowers the solution pH as alkalinity is consumed.^46^ To complete the cycle, the sulfate-bearing effluent from the precipitation reactor was filtered using a 0.45 μm Nylon filter to separate suspended solids and continuously recirculated into the cathode chamber of the electrolyzer. Together, the integrated process described by reactions 6–8 gives reaction 5 overall.

The finite-gap electrolyzer used in these integrated experiments (Figure 2b) consisted of a platinized titanium mesh anode and cathode separated by a FuMA-Tech Fumasep FAS-PET-130 AEM and connected to a galvanostat. Membrane dimensions were approximately 4 cm by 4 cm, and the electrode dimensions were approximately 4 cm by 10 cm separated by the membrane and spacers, such that the total gap distance between electrodes was 3.22 cm. All experiments were conducted using the same piece of membrane, which was rinsed in deionized water and soaked in 1 M Na_2_SO_4_ between experiments.

To start an experiment, the cathode chamber (50 mL) and precipitation reactor (50 mL) were filled with aqueous solution pre-equilibrated with gypsum and atmospheric CO_2_, and then 3.0 g of ground calcium sulfate dihydrate (gypsum) was added to the precipitation reactor. Gypsum powder was prepared by crushing and grinding selenite gypsum (Ward’s Scientific) and sifting to recover the <180 μm size fraction. The initial mass of gypsum used was chosen such that the process rates were independent of the gypsum mass because gypsum rapidly reaches chemical equilibrium with the aqueous solution (Table 1). The initial amount of gypsum did not influence the test results if sufficient gypsum was supplied to avoid complete dissolution of the phase during the experiment. The pre-equilibrated aqueous solution was prepared by mixing doubly deionized water with 5.0 g of powdered gypsum and bubbling the solution with air for 30 min. The equilibrated solution was then vacuum filtered through a 0.2 μm Nylon membrane (Millipore) before filling the precipitation reactor and the cathode chamber. The anode chamber (also 50 mL) was initially filled with doubly deionized water and connected to a recirculating reservoir with an additional volume of 50 mL, such that the both the anode and cathode sides of the system had a total volume of 100 mL of aqueous solution.

**Table 1:** Aqueous Elemental and Thermodynamic Data from Two Time Points during Integrated System Experiments 1–3

		calcium (mM)			sulfate (mM)			precipitation reactor saturation states^a^			
ID	time (min)	anolyte	catholyte	reactor effluent	anolyte	catholyte	reactor effluent	SI calcite	SI aragonite	SI gypsum	*P*_CO_2__ (ppmv)
1	107	0.61	40.1	41.8	2.2	15.2	16.3	3.2	3.0	0.09	3.3 × 103
1	350	0.42	39.9	41.7	4.2	15.4	15.9	3.1	2.9	0.10	5.0 × 10^3^
2	118	1.16	38.1	43.5	4.8	15.1	15.7	3.5	3.4	0.02	9.0 × 10^–2^
2	232	0.28	37.8	40.3	7.9	15.2	15.6	3.5	3.3	0.03	1.9 × 10^–2^
3	50	0.21	15.2	43.5	5.2	13.3	15.1	3.5	3.4	0.04	7.8 × 10^–3^
3	174	0.16	72.4	69.2	17.9	13.1	13.8	3.8	3.6	0.04	9.9 × 10^–4^

a Thermodynamic saturation indices (SI) and equilibrium *P*_CO_2__ values calculated for precipitation reactor solutions.

Chronopotentiometry experiments were conducted for the integrated process under continuous flow, where the galvanostat was powered on at the selected current and the flow was initiated on the cathode and anode sides of the system using two pumps operated at different flow rates. Anolyte solution was recirculated at 40 mL/min to enhance mass transport. The catholyte flow rate of ∼3–6 mL/min was set to allow for an approximately 8–16 min fluid residence time in the precipitation reactor, which allows sufficient reaction to take place so that the pH drop due to calcium carbonate mineral precipitation is easily measurable. The precipitation reactor was continuously mixed with a magnetic stir bar and sparged with atmospheric air using a stainless-steel disseminator, which creates small bubbles that enable rapid CO_2_ dissolution into the aqueous solution. The rate of air sparging was held at 0.3 L air/min in every experiment except Exp. 7 using a mass flow meter to ensure a constant CO_2_ supply (Table 2). Experiment 7 was run for a long duration at a higher air flow rate to generate sufficient solid carbonate products for mineralogical characterization.

**Table 2:** Summary of Measured Rates and Efficiencies of Acid Production and Carbon Mineralization in Integrated System Experiments

ID	initial catholyte	air flow (L/min)	current density (mA/cm^2^)	acid production (10^–6^mol H^+^/min)^a^	carbonation rate, *R*_carb_ (10^–6^mol CaCO_3_/min)	Faradaic efficiency (%)	energy intensity (kW h/mol H_2_SO_4_)
1	0.015 M CaSO_4_	0.3	0.63	3.2 ± 0.8	1.1 ± 0.4	52	0.36 ± 0.10
2	0.015 M CaSO_4_	0.3	1.25	6.7 ± 1.5	3.8 ± 0.6	54	0.40 ± 0.08
3	0.015 M CaSO_4_	0.3	3.13	13.9 ± 4.1	4.8 ± 1.1	39	0.71 ± 0.19
4	0.015 M CaSO_4_	0.3	0.63	3.1 ± 0.4	1.4 ± 0.5	49	0.40 ± 0.04
5	0.015 M CaSO_4_	0.3	1.25	4.3 ± 1.3	2.8 ± 0.6	33	0.72 ± 0.14
6	0.015 M CaSO_4_	0.3	3.13	9.7 ± 2.3	4.4 ± 2.3	31	0.90 ± 0.14
7	0.015 M CaSO_4_	high^b^	3.13	11.7 ± 2.6	12.7 ± 4.2	24	1.00 ± 0.24

a Reported rate of acid production calculated from the measured pH change over time in the recirculating anolyte.

b Air was sparged at a high volumetric flow rate exceeding the capacity of the mass flow controller in Exp. 7, so the flow rate could not be measured.

Replicate experiments were performed at three membrane current densities (3.13, 1.25, and 0.63 mA/cm^2^) to investigate conditions under which the system is kinetically limited by the rate of electrochemical base production (lowest current density) to a condition where base production outpaces alkalinity consumption by gypsum conversion to calcium carbonate (highest current densities). The cell potential was allowed to evolve to maintain a constant current. The time evolution of pH, calcium and sulfate concentrations, and cell potential were monitored throughout the experiment. Fluid samples were taken at three sampling ports (SPs) labeled in Figure 2a: the anolyte (SP1), the catholyte (SP2), and the effluent of the precipitation reactor (SP3).

### Batch Electrochemical Efficiency Tests and Theory

The efficiency of electrolytic sulfate salt splitting to sulfuric acid and base was investigated as a function of sulfate solution composition at elevated (1 M) sulfate concentrations using batch electrolysis experiments with two different electrolyzers: finite-gap and zero-gap. Low current density experiments were conducted with the same finite-gap electrolyzer as used in the integrated experiments, with 1 M Na_2_SO_4_(aq), 1 M MgSO_4_, and 1 M Na_2_SO_4_(aq) equilibrated with gypsum (Table 3, batches 1–3) as sulfate feed solutions to the cathode chamber over a range of current densities up to 18.75 mA/cm^2^. The anolyte and catholyte were rapidly recirculated through the electrolyzer using 100 mL of aqueous sulfate solution prepared using reagent-grade sulfate salts on the cathode side and using 100 mL of doubly deionized Milli-Q water on the anode side. The catholyte was intermittently dosed with dilute sulfuric acid to maintain a moderate pH, mimicking the pH-lowering effect of carbonate mineralization in the integrated process.

**Table 3:** Measured Rates and Efficiencies of Sulfuric Acid Production in Batch Electrolyzer Tests

ID	electrolyzer type	initial catholyte	current density (mA/cm^2^)	acid production (10^–6^mol H^+^/min/cm^2^)	Faradaic efficiency (%)	cell voltage (V)	energy intensity (kW h/mol H_2_SO_4_)
batch 1	finite-gap	1 M Na_2_SO_4_	3.13	1.6 ± 0.2	82	3.2	0.21 ± 0.02
batch 2	finite-gap	1 M MgSO_4_	6.25	2.4 ± 0.2	62	3.4	0.30 ± 0.02
batch 3	finite-gap	1 M Na_2_SO_4_ + gyp^a^	18.8	6.6 ± 2.3	57	3.4	0.36 ± 0.12
batch 4	zero-gap	1 M Na_2_SO_4_	50	34.6 ± 1.7	97	3.2	0.18 ± 0.01
batch 5	zero-gap	1 M Na_2_SO_4_	100	67.9 ± 3.4	95	3.5	0.20 ± 0.01
batch 6	zero-gap	1 M Na_2_SO_4_	150	96.7 ± 4.8	90	4.2	0.25 ± 0.01
batch 7	zero-gap	1 M Na_2_SO_4_	250	173.3 ± 8.7	97	4.6	0.26 ± 0.01
batch 8	zero-gap	1 M Na_2_SO_4_	500	280.0 ± 14	83	5.2	0.34 ± 0.02

a Catholyte solution consists of 1 M Na_2_SO_4_ equilibrated with gypsum.

To test the performance of the system at current densities typical of chlor-alkali (300 mA/cm^2^),^47^ additional batch tests were conducted using a zero-gap water electrolyzer with a surface area of 4.0 cm^2^. Zero-gap electrolyzers are configured with membranes and electrodes in direct physical contact, forming a membrane electrode assembly (MEA). The MEA was constructed using a platinized titanium gas diffusion anode and a nickel foam cathode sandwiching the same type of AEM as used in previous experiments (FuMA-Tech Fumasep FAS-PET-130), with serpentine flow plates on either side to facilitate mixed phase gas–liquid transport out of the electrolyzer. The anolyte (50 mL of 0.1 M Na_2_SO_4_) and catholyte (250 mL of 1 M Na_2_SO_4_) were rapidly recirculated for each applied current density.

The theoretical maximum efficiency for hydrolysis, accounting for the heats of evaporation of H_2_ and O_2_, is 0.08 kW h/mol H_2_SO_4_.^48^ The overall efficiency is a product of the voltage efficiency (*U*_ref_/*U*_cell_) and the current efficiency (1 – *I*_loss_)/*I*, also known as the Faradaic efficiency. The power consumption associated with electrolysis is calculated
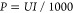
9where *P* is the power in kilowatts (kW), *U* is the cell voltage (V), and *I* is the cell current in amps (A). The energy intensity of sulfuric acid production (kW h/mol H_2_SO_4_) was determined for each experiment by dividing the power by the measured rate of acid production (mol H_2_SO_4_/h).

### Solid and Solution Phase Analysis

For the integrated experiments, subsampled aliquots of aqueous solution were analyzed for pH and sulfate concentration, and the precipitation reactor effluent was also intermittently analyzed for sulfate and calcium cation concentrations. Cation and anion concentrations were measured on filtered samples (0.22 μm Nylon filter, Restek) by ion chromatography using a Metrohm ECO IC with Metrosep cation and anion columns. The aqueous pH was measured using SI Analytics BlueLine pH electrodes calibrated before every experiment using standards at pH 1.68 and 4 for the anolyte and 7 and 10 for the catholyte and precipitation reactor effluent. Solid samples were obtained at the end of experiments by vacuum filtration and air dried at ambient temperature for mineralogical characterization by ATR–FTIR spectroscopy. Spectra were recorded for the solids from 4000 to 525 cm^–1^ by averaging 200 scans at a resolution of 4 cm^–1^ for each measurement (Nicolet iS50). For produced acid concentrations greater than 0.1 M H_2_SO_4_ generated in the zero-gap electrolyzer batch experiments, acid concentrations were quantified by manually titrating 0.1 M reagent-grade sodium hydroxide with a phenolphthalein pH indicator solution. Replicate titrations of a standard were used to quantify the uncertainty on the acid concentration.

### Geochemical Calculations

The aqueous speciation of solutions sampled in the integrated experiments was calculated using the geochemical software PHREEQC (Version 3.3, U.S. Geological Survey). Batch equilibrium calculations were made for the calcium sulfate–calcium carbonate phase assemblage in equilibrium with measured aqueous solution compositions to determine supersaturation indices (SIs) with respect to gypsum and calcium carbonate phases, reported as SI = log_10_(IAP/*K*_sp_), where IAP is the ion activity product calculated in the speciation calculation based on reported elemental concentrations and solution conditions and *K*_sp_ is the thermodynamic solubility product. For calcite and aragonite, IAP = (Ca^2+^)(CO_3_^2–^) with *K*_sp,calcite_ = 10^–8.48^ and *K*_sp,aragonite_ = 10^–8.34^, and for gypsum, IAP = (Ca^2+^)(SO_4_^2–^)(H_2_O)^2^ with *K*_sp,gypsum_ = 10^–4.48^ at 25 °C.

### Kinetic Modeling

The dynamic evolution of pH in the cathode chamber and precipitation reactor can be completely described by contributions from three processes: OH^–^ production at the cathode (*f*_c_, mol OH^–^/min), OH^–^ loss by migration through the AEM or recombination with H^+^ (*f*_loss_), and OH^–^ consumption by mineral carbonation (i.e., reaction 8). Net OH^–^ produced at the cathode (*f*_net,c_ = *f*_c_ – *f*_loss_) enters the precipitation reactor, and a mass balance on pH in the reactor can be written as
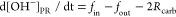
10where *f*_in_ and *f*_out_ represent the flow of OH^–^ into and out of the precipitation reactor, respectively, and *R*_carb_ is the rate of calcium carbonate precipitation (mol CaCO_3_/min). For a volumetric flow rate through the precipitation reactor *Q*_c_ (mL/min), we determine the flux of OH^–^ through the precipitation reactors at time *t* based on the pH of the catholyte (pH_c_; fluid sampled from SP2), *f*_in_(*t*) = *Q*_c_10^–(14–pHc(*t*))^. Similarly, the flux of OH^–^ out of the reactor depends on the pH of the fluid effluent (pH_eff_; fluid sampled from SP2), *f*_out_(*t*) = *Q*_c_10^–(14–pHeff(*t*))^. The rate of mineral carbonation in the precipitation reactor is determined as a function of time by rearranging eq 10
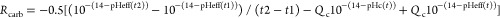
11

## Results and Discussion

### Integrated System Performance

#### Evolution of Fluid Chemistry

**Figure 3:**
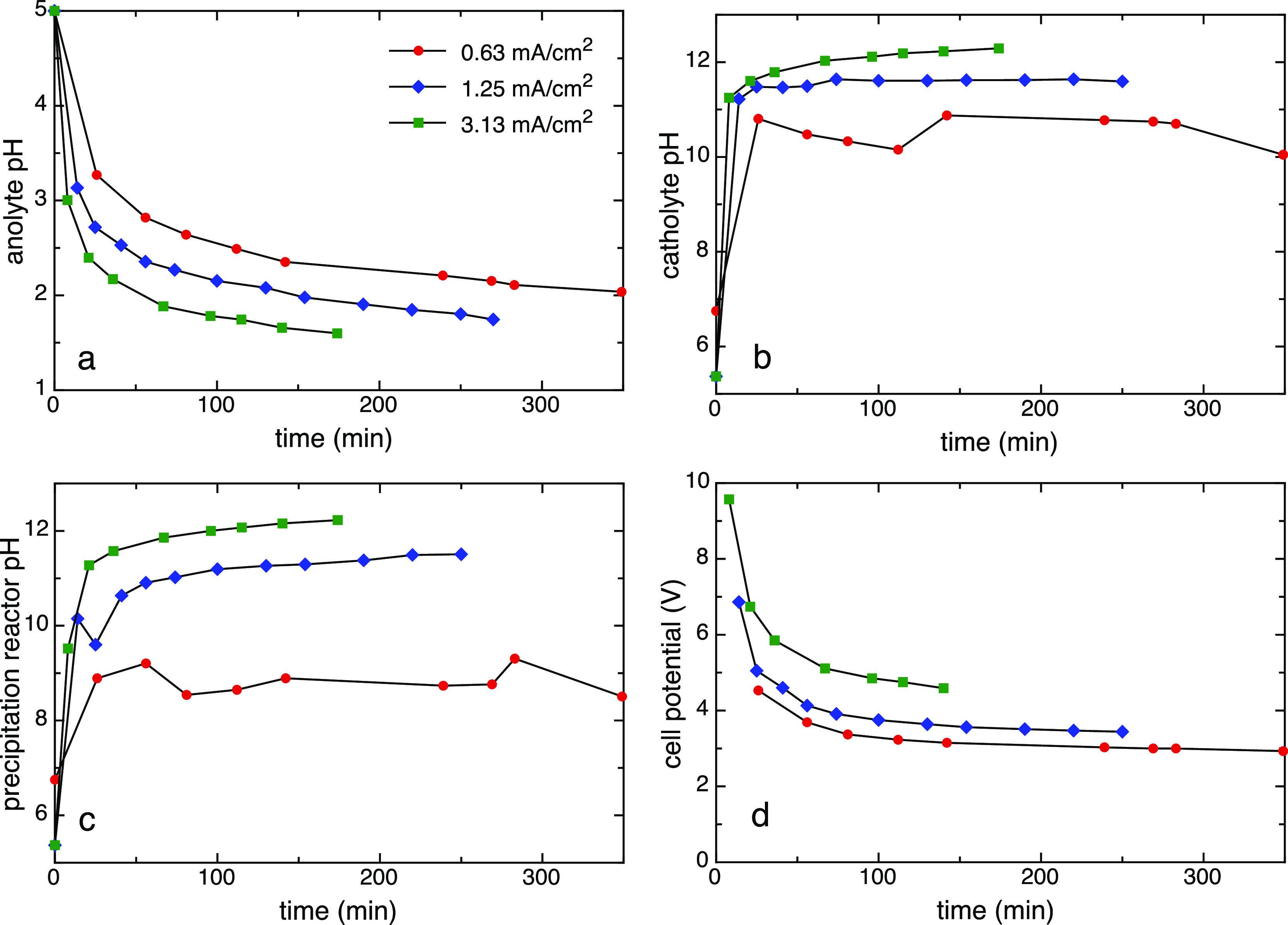
Time evolution of solution pH at sampling points (a) 1 (anolyte), (b) 2 (catholyte), and (c) 3 (precipitation reactor effluent), and (d) electrochemical cell potential data from integrated system experiments 1**–**3 (Table 1). Increasing the operating current yielded faster production of the acid and base with a concurrent increase in the cell potential. Early cell potentials were high due to the high resistance of the initial anolyte, which was pure water, but the voltages evolved toward a steady state.

**Figure 4:**
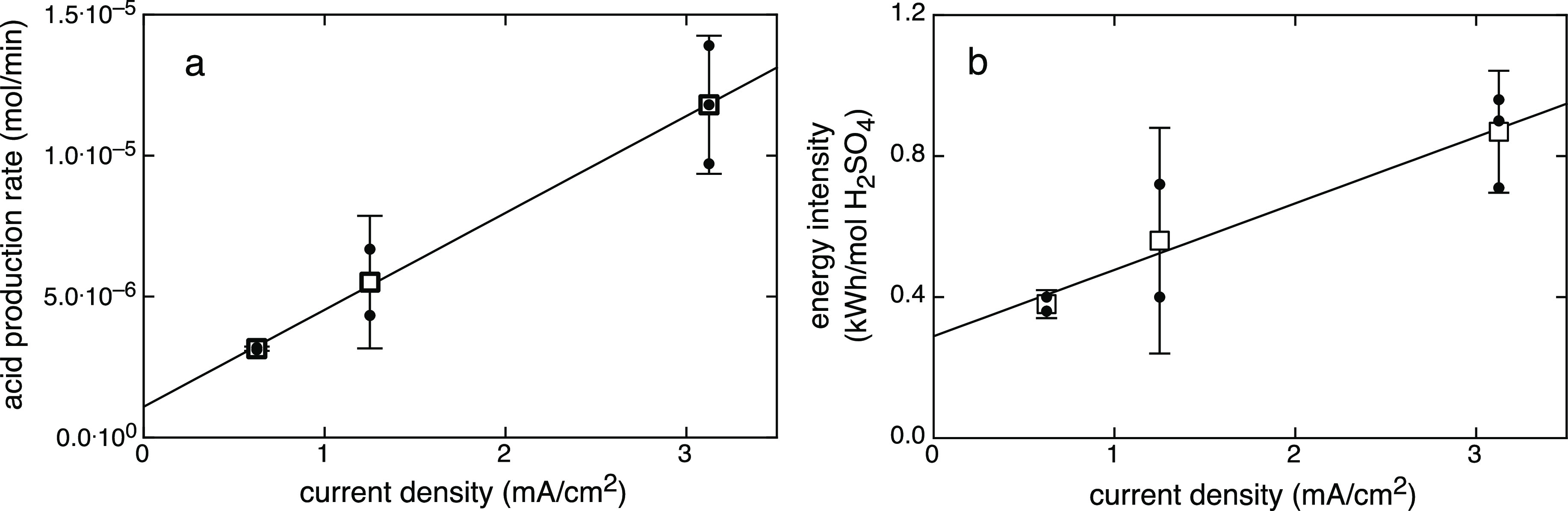
(a) Measured rates of acid production in the integrated system experiments (Figure 2) conducted at a controlled air sparging rate (Exp. 1**–**6). Acid production rates increased linearly with the applied current density. (b) Energy intensity of acid production also increased linearly with the applied current density. Individual experiments are shown as filled circles, and averages of the replicates are shown with 2s.e. uncertainties.

Experiments were conducted over a range of current densities to measure the energy intensity of acid production and the kinetics of CO_2_ mineralization in an integrated system using solid gypsum (CaSO_4_·2H_2_O) as the source of sulfate (Figure 2a; Table 2; reaction 5). Solution pH was measured as a function of time in the anolyte, catholyte, and precipitation reactor effluent (Figure 3a–c), and cell potentials (Figure 3d) were recorded for a set of experiments (Exp 1**–**3; Table 1). Rates of acid production and the corresponding Faradaic efficiencies and energy intensities of acid production were calculated for each experiment (Table 1 and Figure 4a,b). The time-averaged rate of acid production increased linearly with the current density, as expected. The acid production rate was independently determined by measuring the aqueous sulfate concentration of the anolyte solution and subtracting out small contributions from CaSO_4_(aq), and these values are within error of concentrations determined from pH measurements. The measured Faradaic efficiencies ranged from 23 to 54% in these experiments and decreased somewhat with the applied current, while the measured energy intensity increased linearly with applied current (Figure 4b).

Samples of the dissolved aqueous concentrations of calcium and sulfate were also taken at the middle and end of each of experiments 1**–**3 (Table 3). The aqueous concentration of sulfate in the catholyte was controlled by gypsum solubility and was approximately 0.015 M. Sulfate concentrations in the anolyte increased with time due to the accumulation of sulfuric acid in the recirculating solution. Calcium concentrations in the anolyte were low and did not increase significantly over time, indicating that only trace CaSO_4_(aq) species were transported across the AEM into the anolyte, so the produced acid was relatively pure. Geochemical speciation calculations of the measured precipitation reactor compositions indicate that the reactor solutions were close to equilibrium with respect to gypsum in all cases, and the solutions were highly supersaturated with respect to the calcium carbonate polymorphs calcite and aragonite (Table 1). These thermodynamic conditions led to simultaneous gypsum dissolution and calcium carbonate precipitation.

#### Solid Products

**Figure 5:**
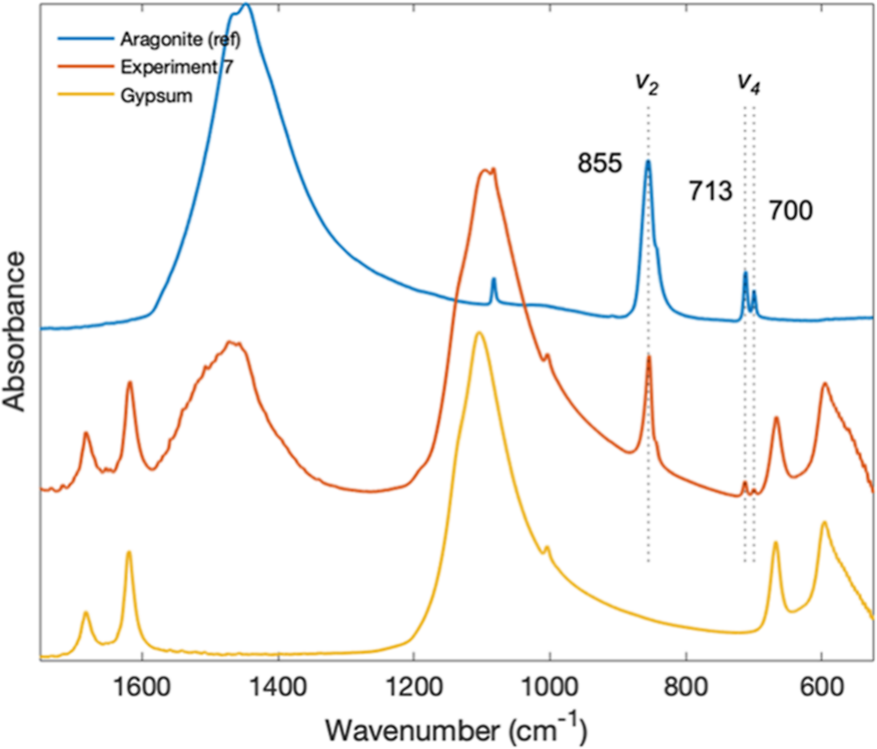
ATR-FTIR spectra of final solids from Exp. 7 along with aragonite (R040078 and RRUFF) and the gypsum starting material. The characteristic peaks of aragonite (855, 713, and 700) are clearly visible in the experimental run products.

The mineralogy of the solid products was determined for an integrated system experiment run for an extended duration (46 h total) at a constant current density of 3.13 mA/cm^2^ (Exp. 7; Table 2). Results of attenuated total reflectance-Fourier-transform infrared (ATR-FTIR) spectroscopy show that gypsum was partially replaced by aragonite (Figure 5). Aragonite is known to form as a transformation product during gypsum replacement by calcium carbonate.^38^ We also observed that the solution in the precipitation reactor was supersaturated with respect to the other crystalline calcium carbonate polymorphs (calcite and vaterite) as well as amorphous calcium carbonate (SI with respect to calcite ∼2 at 25 °C).^49^

### Influence of Solution Composition on Electrolysis Efficiency

**Figure 6:**
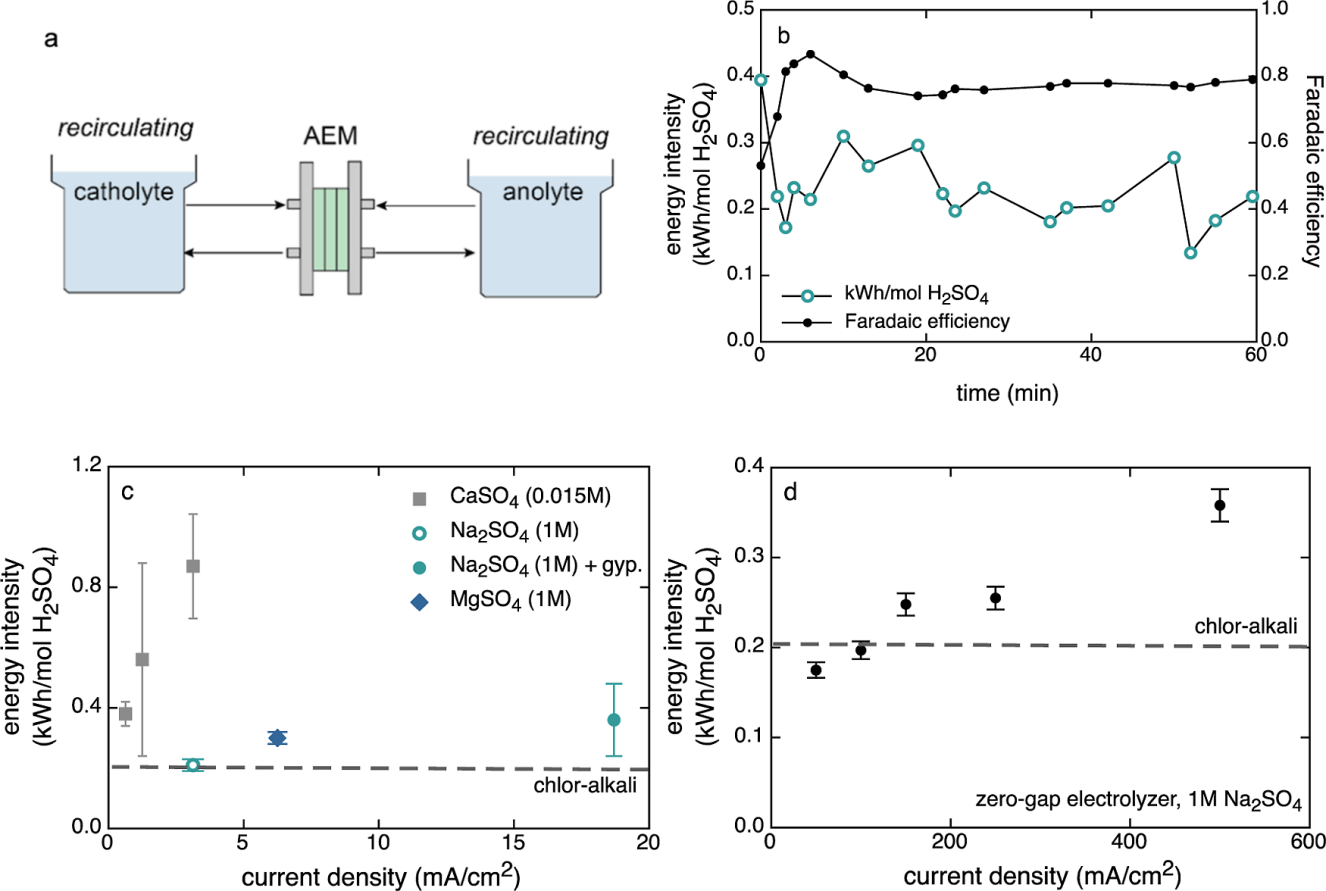
(a) Schematic diagram of the recirculating batch test configuration. (b) Time-resolved energy intensity and Faradaic efficiency data for atest “batch 1” (Table 1) conducted at 3.13 mA/cm^2^ current density in a 1 M Na_2_SO_4_ initial catholyte solution showing the evolution to a high steady-state acid production efficiency after ∼10 min of operation. Sulfuric acid was added to the catholyte solution intermittently to maintain a low concentration of hydroxide relative to sulfate, mimicking the integrated process conditions. (c) Summary data showing the energy intensity of sulfuric acid production in batch experiments compared to the integrated process experiments in gypsum-equilibrated aqueous solutions, all conducted using the finite-gap electrolyzer. (d) Data from the zero-gap electrolyzer indicate that the process can be efficient under industrially relevant current densities.

Batch mode tests were performed to study the dependence of electrolytic acid production efficiency on the composition of the catholyte sulfate feed solution at different current densities. The catholyte feed was composed of either 1 M Na_2_SO_4_, 1 M MgSO_4_, or 1 M Na_2_SO_4_ equilibrated with gypsum (e.g., 0.015 M CaSO_4_(aq)), and doubly deionized water was used as the initial anolyte solution for all experiments. Results of the batch experiments are summarized in Figure 6b–d, and calculated acid production rates, Faradaic efficiencies, cell voltages, and energy intensities of sulfuric acid production are reported in Table 3. The results show that the measured acid production efficiency is relatively insensitive to the cation composition of the solution; however, there is a strong dependence on the aqueous sulfate concentration, indicating that sulfate transport through the membrane is a significant contributor to the cell voltage. The use of a zero-gap electrolyzer at high current density (Figure 6d) actually increased the efficiency of the system relative to the finite-gap electrolyzer at low current density (Figure 6c). The minimum measured energy intensity of acid production was 0.18 ± 0.01 kW h/mol H_2_SO_4_ in 1 M Na_2_SO_4_ at 50 mA/cm^2^ (Figure 6d). This value is comparable to the efficiency of the industrial chlor-alkali process.^50^ Prior to the results reported herein, the best efficiency reported for electrolytic sulfuric acid production was an equivalent to 0.38 kW h/mol H_2_SO_4_ from sodium sulfate, achieved using a two-step bipolar membrane electrodialysis process at a much lower current density.^43^

### Process Kinetics

In the precipitation reactor, the dissolution of gypsum supplies calcium, which reacts with CO_2_ to form precipitated calcium carbonate (Figure 2). The pH in the precipitation reactor is therefore influenced by two competing phenomena: the addition of the base in the catholyte increases pH, and the precipitation of calcium carbonate decreases pH. Under optimal reaction conditions (i.e., CO_2_ flow rate and applied current), these two competing processes should achieve steady-state CO_2_ removal in the reactor.

To determine optimal reaction conditions for our process, integrated experiments were performed at three current densities, 0.63, 1.25, and 3.13 mA/cm^2^, at a constant air flow rate of 300 mL/min. Acid and base production rates increased linearly as applied current was increased (Figure 3a,b). At the same time, we measured an increase in pH in the precipitation reactor as applied current was increased (Figure 3c). These results suggest that increasing current led to an excess of hydroxide ions in the precipitation reactor. These hydroxide ions were not consumed by reaction with CO_2_, and the process was rate-limited by the CO_2_ supply at the higher current densities.

**Figure 7:**
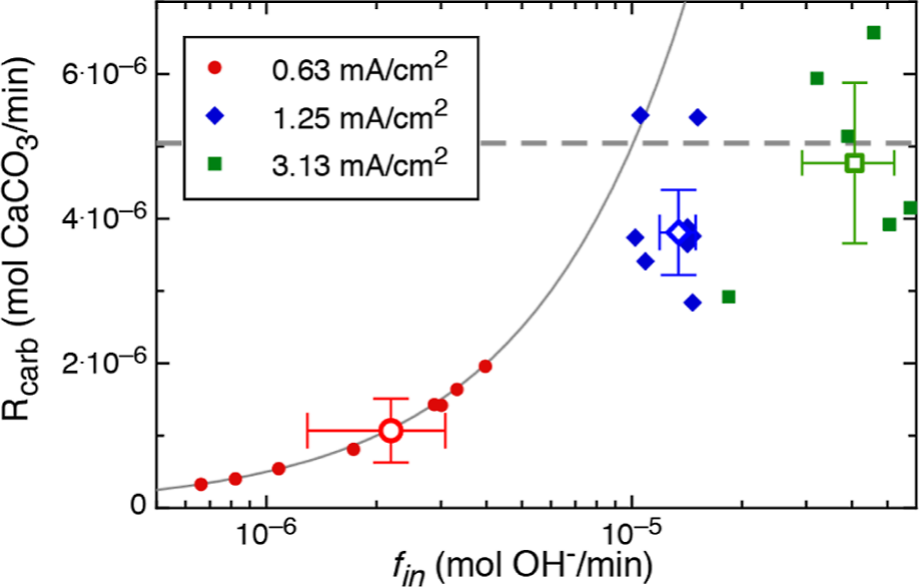
Rate of carbonate precipitation (*R*_carb_; eq 11) is controlled by the OH^–^ supply to the precipitation reactor (*f*_in_) for the lowest current experiment (Exp. 1), as indicated by a fit to the relationship *R*_carb_ = 0.5*f*_in_ (solid line). At higher currents (Exp. 2 and 3), the CO_2_ supply becomes rate-limiting, and the rate of carbonate precipitation evolves toward the CO_2_ flux into the reactor (dashed line).

To confirm the CO_2_ supply rate limitation, the rate of gypsum conversion to calcium carbonate (R_carb_) was quantified based on measured rates of acid and base production (eq 11; Figure 7). The calculated average rates of carbonate mineral precipitation *R*_carb_ = 4.2 ± 0.6 and 4.3 ± 1.2 (×10^–6^ mol/min) were independent of the base supply rate for the 1.25 and 3.13 mA/cm^2^ experiments, respectively (Exp. 2, 3; Figure 7). A precipitation rate that is invariant with the pH and OH^–^ production rate suggests that the supply of CO_2_ was rate-limiting. The mass flux of CO_2_ introduced into the precipitation reactor by air sparging was calculated to be 5.04 × 10^–6^ mol CO_2_/min (assuming a CO_2_ concentration of 380 ppmv) for the constant volumetric flow rate of 300 mL/min, which is close to the measured carbonate precipitation rates for the higher current experiments (1.25 and 3.13 mA/cm^2^). Moreover, the calculated partial pressure of CO_2_ in the precipitation reactor was much less than 1 ppmv in Exp. 2–3 (Table 1), indicating that the solution was highly undersaturated with respect to atmospheric CO_2_. Together, these results confirm that CO_2_ supply was rate-limiting in the 1.25 and 3.13 mA/cm^2^ experiments (Figure 7).

In contrast, at the lowest current density (0.63 mA/cm^2^), the pH in the precipitation reactor evolved toward a steady state of 8–9.5 (Figure 3c; Exp. 1). The fluctuation in pH from 8 to 9.5 can be explained by the observed linear dependence of the carbonation rate on the pH in the precipitation reactor, where *R*_carb_ = 0.5*f*_in_ (Figure 7). The slope of 0.5 is consistent with the stoichiometry of the carbonation reaction (e.g., reactions 4 and 8), where 2 moles of OH^–^ are consumed for every mole of carbonate precipitated. These findings indicate that the process kinetics in the precipitation reactor are completely controlled by the rate of OH^–^ supply for this experimental condition. The lowest current density experiment also yielded the highest Faradaic efficiency of the three currents tested; however, the time-averaged carbonation rate was ∼4 times slower compared to that of the higher current densities. These results highlight the importance of considering both the electrochemical efficiency and the rates of carbonation to optimize the overall process efficiency. A maximum process efficiency will be achieved when the CO_2_ flux is equivalent to the rate of base supply, such that the rate of carbonate precipitation is maximized while maintaining a relatively low steady-state pH in the catholyte to avoid Faradaic losses.

### Energy Intensity Analysis and Implications for Economic Viability

Electrolysis is widely used for industrial acid and base production.^12,51,52^ Water electrolysis is a particularly appealing method for acid generation because the theoretical efficiency of acid generation is high (0.08 kW h/mol H_2_SO_4_ at the thermodynamic limit).^48^ A key challenge with acid generation by water electrolysis, however, is that H^+^ produced at the anode and OH^–^ produced at the cathode can recombine to form water. This recombination process can be reduced by employing an ion-exchange membrane to separate the reactions at the two electrodes. Chlor-alkali electrolysis—the most widely established electrolysis process on an industrial scale—avoids this efficiency loss by producing chlorine gas rather than H^+^ at the anode. As a result, chlor-alkali membrane-cell reactors can achieve high Faradaic efficiencies (>80%) and a minimum energy intensity equivalent to ∼0.2 kW h/mol H_2_SO_4_,^12,50^ although industrial chlor-alkali plants typically operate closer to 0.45 kW h/mol H_2_SO_4_ equivalent.^47^ Similarly, low energy intensities of acid production are achieved here over a range of current densities up to 500 mA/cm^2^ but only when a high sulfate concentration is maintained in the catholyte (Figure 6c–d). The high aqueous sulfate-to-hydroxide ratio in the catholyte minimizes Faradaic losses due to hydroxide ion permeation of the AEM. When more sulfate ions permeate through the AEM than hydroxide ions, the recombination of hydroxide ions and protons in the anolyte is minimized, analogous to chlor-alkali.

Removal of CO_2_ from the air is thermodynamically downhill in basic solutions, but the kinetics depend on the transport and hydrolysis kinetics of CO_2_ at the air-liquid interface. Higher base concentrations accelerate the rate of CO_2_ uptake, and existing air contactor-based CDR approaches use base concentrations on the order of 1 M OH^–^ so that the contactors can be built to a reasonable size for scale-up.^53^ Maintaining a high ratio of aqueous sulfate to hydroxide in the solution feeding the AEM is crucial for maximizing the efficiency of the process demonstrated here, so 1 M hydroxide solutions cannot be efficiently produced in the electrolyzer with an AEM alone. Three-compartment salt-splitting electrolyzers containing both an AEM and a cation exchange membrane (CEM) can simultaneously produce concentrated acids and bases.^54,55^ The three-compartment electrolyzer is expected to improve the Faradaic efficiency of the acid production process because it separates the produced acid and base solutions by a circum-neutral salt solution compartment. However, a typical CEM adds around 0.6 V to the cell voltage,^56^ which, for our process operating at 80% Faradaic efficiency, would increase the electrolysis energy intensity from 0.2 to ∼0.25 kW h/mol H_2_SO_4_.

### Improvements to Efficiency

Collectively, these data indicate that sulfuric acid production by water electrolysis in a membrane-cell can operate at efficiencies on par with the best-performing chlor-alkali reactors in terms of the energy intensity of acid and base production. However, to be industrially viable, it is necessary to achieve low cell voltages and high current efficiencies at high current densities (>100 mA/cm^2^) for long periods of time (months to years). Simple improvements in electrolyzer design, including decreasing the gap between electrodes, employing porous electrodes with high surface areas, and flowing solution directly at the electrode surface using flow fields, can maximize efficiency.^57^ These improvements reduce voltage losses caused by electrolyte resistance and increase energy efficiency at high current densities, as shown in Figure 6d. Anion-exchange membranes with weakly basic anion-exchange groups have been shown to limit proton leakage.^58−61^ These AEMs could enable the production of higher concentrations of sulfuric acid by minimizing Faradaic losses; however, cell voltage may also increase due to a reduction in sulfate transport through the AEM.

The accumulation of mineral scale (i.e., solid products) on the membrane and electrodes can lead to voltage increases over time by reducing the catalytic surface area and blocking charge transport. However, mineral scaling can be mitigated by brine treatment to reduce or eliminate Mg or Ca from the electrolyzer feed solution,^47^ which would maintain a low aqueous supersaturation index with respect to scale-forming phases. In calcium solutions, for example, minimizing calcite supersaturation in the precipitation reactor helps prevent uncontrolled nucleation, driving precipitation onto pre-existing seed mineral surfaces. Operating the precipitation reactor at a higher temperature relative to the catholyte can also reduce scale formation in the electrolyzer. At higher temperatures, the solubility of calcium carbonate decreases and more solid products are precipitated, while at the same time, the solubility of gypsum increases to provide higher sulfate concentrations. Mineral scales formed by sparingly soluble calcium and magnesium hydroxy-carbonates can be managed by intermittently applying a pulse-current or reversing the polarity of the electrodes to remove any solids formed in the electrolyzer.^62^ The scaling of gypsum or other sulfate phases is not anticipated because they are at equilibrium or undersaturated in solution.

### Achieving Large-Scale Carbon Dioxide Removal

Gigaton-scale CO_2_ removal and permanent sequestration is now crucial for limiting global warming to relatively safe levels.^3^ Minimizing the cost while ensuring the integrity and permanence of removal through adequate monitoring and verification are critical for voluntary and compliance markets to be established.^5^ The total energy requirement of the process described in this article is 4.2 MW h/ton CO_2_ sequestered (15.2 GJ/t), assuming an energy intensity of acid production of 0.2 kW h/mol H_2_SO_4_ and that energy is recovered by hydrogen combustion in a fuel cell with 60% efficiency (Supporting Information).^63,64^ Improvements to the electrolyzer could eventually reduce the energy intensity to approach the energy intensity of polymer electrolyte membrane (PEM) electrolysis (0.1 kW h/mol),^48^ such that the minimum theoretical energy of CDRS by this process is 1.9 MW h/t CO_2_ (6.8 GJ/t). For comparison, most leading technologies being deployed for direct air capture and sequestration require at least 11 GJ/t CO_2_, and much of this is in the form of heat.^53,65,66^ The improvement of electrolyzer performance to 0.15 kW h/mol H_2_SO_4_ will make this process equally efficient as leading CDR technologies, while at the same time producing recycled sulfuric acid for use in the mining industry and solid carbonate products that can be used as components of low carbon-intensity cement (i.e., “green cement”).^67−69^

Sulfuric acid is the most widely used inorganic chemical in the world and is mainly used in phosphate fertilizer production and in ore and tailings processing. Conventional sulfuric acid is mainly produced by the oxidation of elemental sulfur that is typically derived from fossil fuels, although the smelting of sulfidic ores also contributes significantly to the global supply.^30^ Shortages of the acid have been projected by 2040 due to the increased demand for critical element extraction paired with a tightening sulfur supply as fossil fuel refining declines.^70^ A basic techno economic model was developed to evaluate the economic viability of electrolytic sulfuric acid production with DACS. The cost of a polymer electrode membrane electrolyzer for hydrogen generation is approximately $430/kW.^64^ Assuming a twofold higher capital cost for electrolyzers, an energy intensity of acid production of 0.2 kW h/mol H_2_SO_4_, and an electricity cost of $0.03/kW h, the total cost to produce electrolytic sulfuric acid is $112/ton, which is on par with historical commodity prices. Pumping of water and air contributes moderately to the overall process cost (∼$11/ton sulfuric acid; Supporting Information). Several factors are expected to improve the economics of adopting this process over time. Sulfur shortages are anticipated in the coming decades,^70^ and the cost of managing sulfate wastes in the mining and fertilizer industries will continue to increase over time. The generation of revenue from saleable products including green hydrogen, CO_2_ removal and sequestration credits, and sales of precipitated calcium carbonate can improve the unit economics further, but even without this, electrolytic sulfuric acid production with carbon mineralization can be economically viable for a variety of applications.

To achieve gigaton-scale carbon sequestration, it will be necessary to replace the supply of sulfuric acid to major industrial processes that liberate CO_2_-reactive elements, such as P fertilizer production and acid extraction of critical elements from ultramafic rocks. Today, more than 700 sulfur burner plants supply sulfuric acid for extractive processes. Phosphoric acid production is globally the largest consumer of sulfuric acid (reaction 3) and has generated gigatons of PG. While mined gypsum is a valuable commodity, PG waste cannot be used in many countries including the United States because it contains trace amounts of naturally occurring radioactive elements. Conversion of PG waste into carbonate minerals during phosphate fertilizer production can permanently sequester 50**–**75 Mt of CO_2_ every year, while at the same time ending the production of sulfate waste. Many of the trace element constituents of PG, including rare earth elements, uranium, and other metals, can be valorized following selective recovery from PG.^32,71^ Radium (Ra^2+^) is the main source of ionizing radiation in PG,^71,72^ and this species partitions much more weakly into carbonates than sulfates due to an ionic size mismatch. Thus, the conversion of PGs into calcium carbonate can reduce the environmental toxicity and associated waste management costs of phosphate fertilizer production. Sulfuric acid reconcentration may be required for phosphoric acid production, but industrial sulfuric acid concentration systems are commercially available.^73,74^

The mining of energy critical elements (e.g., Li, Ni, and Co) is dramatically increasing to facilitate the transition of the global energy infrastructure away from fossil fuels. By 2040, production of Li, Ni, and Co must increase by more than five times (relative to 2020) to meet a reasonable sustainable development scenario.^16^ Many of these elements can be extracted from rocks with substantial ANP, which means that increased critical element mining could enable gigaton-scale CO_2_ removal from the atmosphere.^13−15,75^ By incorporating enhanced rock weathering into the process proposed in this article, it is feasible to achieve gigaton per year CDR and sequestration that is affordable and permanent, while at the same time increasing critical element yields from mine tailings. Tailings are typically already very fine-grained, enabling fast reaction kinetics, so no additional comminution or pretreatment is likely required to use these materials as feedstocks to the process.^76^ Scaling-up to more than 100 Mt per year of CO_2_ sequestration by this process will require the construction of several hundred plants of a size similar to world-scale sulfur burner plants^30^ (e.g., 2000–4000 t/d sulfuric acid production), which is feasible to achieve over the course of decades as existing sulfur burners reach their useful lifespans.

As global industries begin to prioritize lowering carbon emissions and renewable electricity becomes more readily available, new opportunities arise to replace legacy chemical production methods with sustainable electrified processes. Here, the proof-of-concept was established for a process to replace production of the most-used inorganic chemical in the world, sulfuric acid, while permanently sequestering CO_2_ from the air at a mole-for-mole basis. Approximately half a ton of CO_2_ can be mineralized by this process per ton of sulfuric acid. Environmental co-benefits of the process include the ability to recycle sulfate waste and to co-produce green hydrogen. The efficiency and energy intensity of this process compare favorably with established industrial electrolytic processes for chemical production. The global adoption of this process in favor of the traditional sulfur oxidation process has the potential to sequester more than 100 Mt of CO_2_ per year based on the current acid usage of ∼250 Mt/yr,^21^ which amounts to 1**–**5% of the global target for carbon removal technologies. Much larger, gigaton-scale removals are achievable by using electrolytically recycled sulfuric acid to enhance weathering of basic and ultramafic mine tailings produced during the extraction of elements essential for the renewable energy transition.

## Supporting Information Available

Background information on silicate weathering, carbon dioxide mineralization, the Urey cycle; and additional information used to complete the energy balance and techno-economic calculations cited in the main text (PDF)
