# Supplementary material for: Electrolytic Sulfuric Acid Production with Carbon Mineralization for Permanent Carbon Dioxide Removal
Source: ACS Sustain Chem Eng. 2023 Mar 13;11(12):4800–12. doi: 10.1021/acssuschemeng.2c07441 (PMC10052359; doi:10.1021/acssuschemeng.2c07441)
Supplement: Supplementary file 1 — sc2c07441_si_001.pdf [file sc2c07441_si_001.pdf]

Supporting Information for

Electrolytic sulfuric acid production with carbon mineralization for permanent carbon dioxide removal

Laura N. Lammers[1,2*], Yanghua Duan[3], Luis Anaya[1], Ayumi Koishi[4], Romario Lopez[1], Roxanna Delima[2], David Jassby[5], David L. Sedlak[3]

[1]Department of Environmental Science, Policy, and Management, University of California, Berkeley, CA 94720, USA
[2]Travertine Technologies, Inc., Boulder, CO 80301, USA
[3]Department of Civil and Environmental Engineering, University of California, Berkeley, CA 94720, USA
[4]Energy Geoscience Division, Lawrence Berkeley National Laboratory, Berkeley, CA 94720, USA
[5]Department of Civil and Environmental Engineering, University of California, Los Angeles, CA 90095, USA
*corresponding author, laura@travertinetech.com

Number of pages: 2
Number of tables: 0
Number of figures: 0

Chemical weathering and mineral carbonation

Chemical weathering of silicates occurs by protonation and hydrolysis, where dissolution of silicates neutralizes acidity and releases carbon dioxide reactive elements, especially Mg^{2+} and Ca^{2+}. For the important rock forming mineral forsterite olivine, an example chemical weathering reaction can be written,

$$\frac{1}{2} Mg_2SiO_4(s) + 2H^+(aq) \rightarrow \frac{1}{2} SiO_2 \cdot 2H_2O(s) + Mg^{2+}(aq). \quad [S1]$$

At the same time, reaction of atmospheric carbon dioxide with meteoric water produces dissolved inorganic carbon (DIC) species bicarbonate (HCO_3^-) and carbonate (CO_3^{2-}):

$$CO_2(g) + H_2O(l) \rightarrow H^+(aq) + HCO_3^-(aq) \rightarrow 2H^+(aq) + CO_3^{2-}(aq). \quad [S2]$$

These DIC species can react with carbon dioxide reactive elements to produce solid carbonate minerals, which are stable over geologic timescales, e.g.:

$$Mg^{2+}(aq) + CO_3^{2-}(aq) \rightarrow MgCO_3(s). \quad [S3]$$

The overall chemical weathering reaction drives the solution in equilibrium with atmospheric carbon dioxide into saturation with respect to carbonate minerals including polymorphs of calcium carbonate (e.g., calcite, aragonite, vaterite) as well as variably hydrated magnesium carbonate phases (e.g., magnesite, nesquehonite).

The overall mineral carbonation reaction for forsterite is given,

$$\frac{1}{2} Mg_2SiO_4(s) + CO_2(g) + H_2O(l) \rightarrow \frac{1}{2} SiO_2 \cdot 2H_2O(s) + MgCO_3(s). \quad [S4]$$

The process of rock weathering to buffer the atmospheric CO_2 concentration is known as the Urey Cycle[1].

Energy Balance and Economic Calculations

To estimate the energy intensity and cost of a scaled-up process for electrolytic sulfuric acid production and recycling with direct air carbon dioxide capture and mineral carbon sequestration, we identified the process steps associated with significant energy consumption. These include (1) electrolysis, (2) water pumping, and (3) air blowing. In the cases where concentration of sulfuric acid is required for the desired application, the energy intensity of that process was also considered.

Electrolysis is the most energy intensive aspect of electrolytic sulfuric acid production. For one metric ton of sulfuric acid produced at an energy intensity of 0.2 kWh/mol H_2SO_4, the energy requirement is 2.04 MWh/t sulfuric acid. Blowing of air in an air contactor to supply aqueous carbonate to the precipitation reactor requires approximately 0.1 MWh/t sulfuric acid. Pumping aqueous solutions and mixing slurry requires approximately 0.28 MWh/t sulfuric acid. Evaporative concentration from 10.7 wt. % (1 M H_2SO_4) to 80 wt.% H_2SO_4 by three-effect

evaporation is approximated as one third of the latent heat of water evaporation or 1.8 MWh/t sulfuric acid.

Combining these processes, the total electrical energy requirement is estimated to be 2.4 MWh/t dilute sulfuric acid produced. At a renewable electricity price of $30.00/MWh, pumping of air and water will cost $11.40/t H_2SO_4. The overall cost of energy including electrolysis is $72/ton sulfuric acid without evaporative concentration. Assuming (1) the electrolyzer capital cost is roughly four times the cost of a polymer electrolyte membrane electrolyzer target price ($430/kW),[2] and (2) the total plant capital cost is roughly double the electrolyzer cost and lasts roughly10 years, capital contributes around $80/t H_2SO_4. The total sulfuric acid price without concentration is approximately $112/t.

References

(1) Sleep, N. H.; Zahnle, K. Carbon dioxide cycling and implications for climate on ancient Earth. *Journal of Geophysical Research: Planets* **2001**, *106* (E1), 1373-1399.
(2) Department of Energy, O. o. E. E. R. E. *DOE Technical Targets for Hydrogen Production from Electrolysis*. 2018. https://www.energy.gov/eere/fuelcells/doe-technical-targets-hydrogen-production-electrolysis (accessed 2021 September).
